# Hypokalaemia and cardiac arrest complicating vancomycin and furosemide therapy: a case report

**DOI:** 10.4076/1757-1626-2-8244

**Published:** 2009-07-31

**Authors:** Keith Siau

**Affiliations:** Department of Medicine, Musgrove Park HospitalTaunton, TA1 5DAUK

## Abstract

**Introduction:**

Hypokalaemia is an unrecognised complication of vancomycin therapy.

**Case presentation:**

We present a 68-year-old female with an infected hindquarter amputation site who had incurred severe hypokalaemia with recurrent episodes of cardiac arrest requiring cardioversion shortly after commencing intravenous vancomycin therapy. The cause of hypokalaemic cardiac arrest was thought to be due to administration of low dose furosemide, however, holding potassium-wasting diuretics did not restore normal serum potassium concentrations. After an extended period of conservative management with potassium supplementation, spironolactone therapy and cautious serum electrolyte monitoring, cessation of vancomycin led to the complete resolution of hypokalaemia.

**Conclusion:**

Clinicians should be aware that vancomycin therapy, even in the presence of normal renal function, may be a reversible cause of severe hypokalaemia.

## Introduction

Severe hypokalaemia associated with antibiotic use is rare and is a recognised complication of dicloxacillin and aminoglycosides, especially in conjunction with renal or hepatic insufficiency. However, there have been no previous reports associating vancomycin use with profound hypokalaemia.

## Case presentation

A 77-year-old Caucasian female was admitted under the orthopaedic team for rehabilitation 2 months following a left sided hindquarter amputation. There was no radiological evidence of metastatic disease. Her significant past medical history included moderate degree hypertension and mild left ventricular failure with an ejection fraction of 55%. Her medications included chronic use of multiple anti-hypertensives including ramipril, atenolol and bendroflumethiazide, along with high dose opioids for pain control. She had previously been on multiple courses of 40 mg oral furosemide for peripheral oedema.

Due to prolonged non-healing of her amputation site, intravenous vancomycin (1 g twice daily) was commenced empirically for suspected localised wound infection, with serum vancomycin levels monitored routinely. She was otherwise well without features of sepsis. Her serum potassium levels dropped from 4.2 mmol/L to 2.9 mmol/L one week after starting vancomycin, and oral potassium (Sando-K) was promptly initiated and bendroflumethiazide stopped. No other changes to her medications had been made during this time. There was no evidence to suggest additional potassium loss from the wound or from the gastrointestinal tract. The patient was subjectively well with good oral intake. After prolonged recumbency, the patient developed small bilateral pleural effusions and peripheral oedema. Administration of low dose oral furosemide (20 mg) resulted in a significant drop in serum potassium from 4 mmol/L to 1.7 mmol/L overnight, which induced pulseless ventricular tachycardia (VT) prompting cardioversion. Other biochemical parameters including sodium (138 mmol/L), corrected calcium (2.4 mmol/L) were normal. She was mildly hypomagnesaemic (0.5 mmol/L), which was corrected. Despite aggressive intravenous potassium replacement, she developed another episode of pulseless VT arrest with potassium level of 2.8 mmol/L. She was again resuscitated, with furosemide being converted to spironolactone, a potassium-sparing diuretic. When stabilised with intravenous potassium supplementation, and then onto 2 Sando K+ three times daily. Serum investigations performed to explore the possibility of Cushing’s syndrome and Conn’s disease were unremarkable. By relating potassium levels with her medication history, potassium depletion seemed to correlate with vancomycin therapy, and when combined with furosemide, had led to a dramatic fall in serum potassium.

Spironolactone was replaced with amiloride to promote diuresis, which led to mild hyperkalaemia. After finishing a prolonged course of vancomycin, serum levels had normalised and amiloride was stopped. Serum potassium levels remained stable thereafter.

## Discussion

Although hypokalaemia following aminoglycoside use is well recognised [1], this has not previously been reported in conjunction with vancomycin use. Severe hypokalaemia has also been reported in conjunction with dicloxacillin use [2]. Within the British National Formulary [1], hypokalaemia secondary to vancomycin use is not a recognised feature. Furthermore, there is no evidence in the literature to suggest that vancomycin may potentiate the effects of furosemide.

**Figure 1.**
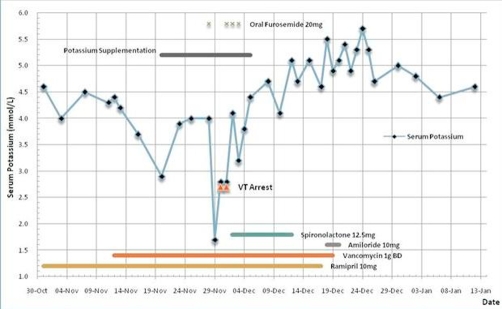
Timeline of events demonstrating fluctuations in serum potassium in our patient.

Three observations could be made from Figure 1: 1) Vancomycin administration *per se* may have resulted in the subacute development of hypokalaemia, 2) concomitant use of furosemide and vancomycin may have resulted in potassium depletion which preceded the hypokalaemic VF arrests, 3) withdrawal of vancomycin resulted in the normalisation of serum potassium.

Electrolyte imbalances secondary to loop diuretic use is well recognised. We appreciate that the use of furosemide invariably confounds the picture, and some observers may bring our findings into question. We argue that, although low doses of oral furosemide may cause hypokalaemia, it seldom causes hypokalaemia severe enough to precipitate cardiac arrest, especially if the patient had tolerated heavier dosages of the drug previously. There had been no additional losses from her wound or gastrointestinal tract, and the patient’s food intake had not been compromised. The patient’s fluctuations in serum potassium had clearly correlated with vancomycin administration, which had achieved therapeutic serum concentrations. We postulate that the mechanism of vancomycin-induced hypokalaemia, as is the case with aminoglycoside-induced hypokalaemia, is through renal wasting. We regret that urinary potassium had not been measured prior to the window between vancomycin administration and spironolactone administration. We suspect that if this had been done, urinary potassium concentrations may have been high, thus reflecting suspected renal losses. A larger study is required to confirm our findings.

## Conclusion

We report a potential case of vancomycin-induced hypokalaemia which had resulted in cardiac arrest. We therefore recommend for electrolytes to be monitored in patients receiving vancomycin therapy, especially those with concomitant diuretic therapy.

## Abbreviation

VT: ventricular tachycardia
